# Building Bridges between People with Stroke, Families, and Health Professionals: Development of a Blended Care Program for Self-Management

**DOI:** 10.3390/jcm13010300

**Published:** 2024-01-04

**Authors:** Carla Mendes Pereira, Mara Matos, Daniel Carvalho, Patricia Macedo, José M. Calheiros, Janice Alves, Luís Paulino Ferreira, Teresa L. Dias, Rui Neves Madeira, Fiona Jones

**Affiliations:** 1 Department of Physiotherapy, School of Health, Polytechnic University of Setúbal, 2910-761 Setúbal, Portugal; mara.matos@ess.ips.pt (M.M.); teresa.dias@ess.ips.pt (T.L.D.); 2 Comprehensive Health Research Centre (CHRC), NOVA University of Lisbon, 1150-082 Lisbon, Portugal; 3 Local Health Unit Litoral Alentejano (ULSLA), 7540-230 Santiago do Cacém, Portugal; daniel.carvalho@ulsla.min-saude.pt; 4 Research Center for Engineering a Sustainable Development (Sustain.RD), Setúbal School of Technology, Polytechnic University of Setúbal, 2910-761 Setúbal, Portugal; patricia.macedo@estsetubal.ips.pt (P.M.); rui.madeira@estsetubal.ips.pt (R.N.M.); 5 Center of Technology and Systems (UNINOVA-CTS), NOVA School of Science and Technology, 2829-516 Caparica, Portugal; 6 Institute for Research, Innovation and Development (FP-I3ID), University Fernando Pessoa, 4249-004 Porto, Portugal; jcalheiros@ufp.edu.pt; 7 Neurology Department, Setúbal Hospital Centre, 2910-446 Setúbal, Portugal; janice.alves@chs.min-saude.pt; 8 Department of Psychiatry and Mental Health, Setúbal Hospital Centre, 2910-446 Setúbal, Portugal; luis.ferreira@chs.min-saude.pt; 9 NOVA Laboratory of Computer Science and Informatics (NOVA LINCS), NOVA School of Science and Technology, NOVA University of Lisbon, 2829-516 Caparica, Portugal; 10 Population Health Research Institute, St George’s, University of London, London SW17 ORE, UK; fjones@sgul.ac.uk

**Keywords:** self-efficacy, self-management, stroke, change behavior, co-production, eHealth, usability, quality, mHealth

## Abstract

Evidence-informed interventions for stroke self-management support can influence functional capability and social participation. People with stroke should be offered self-management support after hospital discharge. However, in Portugal, there are no known programs of this nature. This study aimed to develop a person-centered and tailored blended care program for post-stroke self-management, taking into account the existing evidence-informed interventions and the perspectives of Portuguese people with stroke, caregivers, and health professionals. An exploratory sequential mixed methods approach was used, including qualitative methods during stakeholder consultation (stage 1) and co-production (stage 2) and quantitative assessment during prototyping (stage 3). After ethical approval, recruitment occurred in three health units. Results from a literature search led to the adaptation of the Bridges Stroke Self-Management Program. In stage one, 47 participants were interviewed, with two themes emerging: (i) Personalized support and (ii) Building Bridges through small steps. In stage two, the ComVida program was developed, combining in-person and digital approaches, supported by a workbook and a mobile app. In stage three, 56 participants evaluated prototypes, demonstrating a strong level of quality. Understandability and actionability of the developed tools obtained high scores (91–100%). The app also showed good usability (A-grade) and high levels of recommendation (5 stars).

## 1. Introduction

Despite global advances in acute care and a decline in incidence rates, stroke remains the second-leading cause of death and disability combined worldwide [1]. In 2017, in Europe, there were 9.5 million stroke survivors, 7 million disability-adjusted life-years (DALYs) lost due to stroke, and estimated costs at €45 billion [2]. Accordingly, many people with stroke remain living with complex, long-lasting consequences, including physical, cognitive, and psychosocial impairments, resulting in participation restrictions and a high prevalence of unmet needs [3,4].

The impact of a stroke is particularly evident during the hospital-home transition, a period marked often by uncertainty, fears and loss of autonomy experienced by both people with stroke and their informal caregivers/family [5,6]. The increased emphasis on an early-supported discharge model of care reinforces the importance of coordinated discharge plans and the engagement of both people with stroke and caregivers to guarantee a successful transition to the community [5,7]. Although people tend to consider discharge timing as appropriate and the hospital-home transition being eased by early supported discharge [8], problems are often reported regarding the transition to community services. Factors, such as the negative impact of the lack of continuity in care and services’ inactivity (e.g., lack of active follow-up, delayed access to community services), are highlighted as having a negative impact on both people with stroke and informal caregivers [8,9].

However, personalized support strategies used by healthcare professionals can influence adjustment and self-efficacy after stroke by enabling collaboration and involvement in ongoing plans [10]. This can build a relationship between people with stroke, their caregivers, and healthcare professionals that does not promote dependency but promotes greater self-management and choice about what is most important [9,10].

According to the European Stroke Action Plan (ESAP) for 2030, all people with stroke with residual impairments at the moment of hospital discharge should be provided with effective self-management support [11]. However, in Portugal, according to previous findings from a longitudinal study of coping and adjustment after stroke, both people with stroke and informal caregivers reported continued dependency on professionals’ counseling and advice [12]. Therefore, decision-making often occurs without their involvement and many challenges are encountered while pursuing a balance between encouraging people to take control and guiding their decisions [13]. The Portuguese Health Plan recognizes the need for a patient-centered approach and the importance of training for professionals that is aimed at empowering patients [14]. Therefore, a paradigm shift is needed in order to provide people with the support needed to develop the skills, knowledge and self-efficacy to manage their daily lives and to actively collaborate with health professionals, aiming for a mutually committed person-professional partnership [15].

International stroke guidelines [16,17,18] and the World Stroke Organization [19] recommend self-management support as a core part of stroke rehabilitation. This support is based on the understanding that a person living with a long-term condition should be at the center of managing life with the condition and not the healthcare services. The principles of self-management support focus on collaborative partnerships between individuals and health and social care professionals, seeking to facilitate behavior change rather than providing a purely educational program [20]. This can be achieved by providing people with the support needed to develop the skills, knowledge and confidence to manage clinical and emotional aspects of their chronic condition [10] through regular assessment of progress, goal setting, and problem-solving support [20]. Results from systematic reviews assessing a wide range of interventions following stroke reported improvement in physical and function domains such as participation and reintegration in the community, quality of life and self-efficacy, as well as in reducing hospital readmission rates and healthcare utilization [10,21,22]. However, in Portugal, there are no known programs of this nature for people with stroke. Taking into account the current evidence-informed interventions, an understanding of the contextual needs is important when developing an existing feasible intervention [23]. This includes a greater understanding of cultural differences from where the intervention has been designed and evaluated [24].

Furthermore, the rather rapid adaptation to digital care intensified and accelerated during the COVID-19 pandemic, has uncovered many possibilities. The potential for digital interventions to support self-management approaches in chronic conditions has been increasingly recognized [25]. Previous findings indicate that some digital interventions facilitate self-management of the condition [25,26], with chronic people becoming more aware of their condition, more capable of making health decisions and engaging in meaningful discussions as equals with the healthcare professional. The use of eHealth interventions may have the potential to empower the person with stroke and informal caregivers to prevent “abandonment” feelings, aid in anxiety management at discharge and provide a monitoring platform for those who need it [27]. Although rapid changes to digital care are evolving, the research on the benefits of self-management support in stroke care is scarce. Nonetheless, there is emerging evidence regarding the effectiveness of a theory-based digital self-management intervention in improving levels of depression, anxiety, fatigue and self-efficacy among people with neurological disorders [28]. However, these findings are not tailored for people with stroke and their caregivers. Research on stroke, particularly regarding the application of novel digital interventions, has predominantly concentrated on secondary prevention (e.g., [29,30,31]). Moreover, while recent studies have investigated the potential of digital tools to enhance post-stroke self-management interventions, the practical implementation of these digital components has been largely confined to electronic messaging, smartphone push notifications, and video conferencing [32,33,34,35].

To our knowledge, limited research has been conducted on the interventions that integrate both in-person and eHealth self-management solutions. For instance, Cameron et al. [35] and Kamoen et al. [29] developed hybrid programs; however, in both, the degree to which eHealth capabilities were used was limited, as none of these systems took full advantage of interactive features with digital components or user input.

Given the existing evidence for in-person stroke self-management interventions, promising results of some digital interventions, and the potential solutions of eHealth because of their low cost and adaptability, combining both is worth consideration, as long as they tailor to the profile of users, their needs and preferences. The main aim of this study was to explore the potential of evidence-informed interventions for self-management support after stroke. Following this, to adapt existing interventions and add new components based on the needs, concerns, and motivations of Portuguese people with stroke, along with insights from informal caregivers and health professionals, in order to develop a person-centered and tailored blended care program for post-stroke self-management during the rehabilitation process. After an iterative co-production process, this study also aimed to assess the usability, quality, understandability, and actionability of the developed intervention.

## 2. Materials and Methods

### 2.1. Study Design, Setting, and Participants

**Figure 1:**
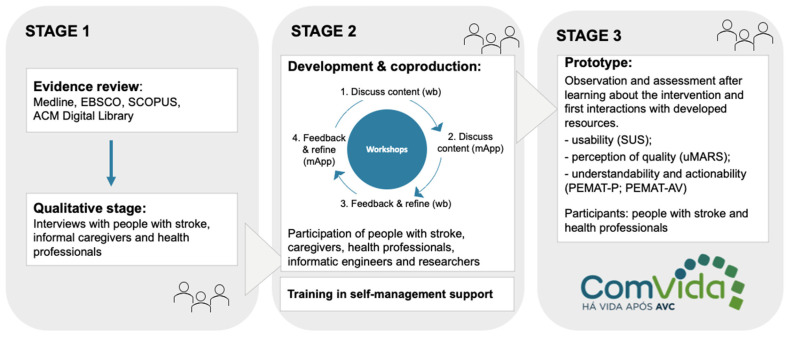
Description of the study stages. Wb = workbook; mApp = mobile app; PEMAT-P = Patient Education Materials Assessment Tool—for Printable materials; PEMAT-AV = Patient Education Materials Assessment Tool—for Audiovisual materials; uMARS = Mobile App Rating Scale-user version; SUS = System Usability Scale.

An exploratory sequential mixed methods approach was used, with both qualitative and quantitative methods helping to inform adaptation and development process decisions in a three-stage framework [23,36,37]. The three stages were: (i) evidence review and stakeholder consultation through a qualitative exploration of the needs, concerns, motivations, and useful strategies for self-management support after stroke in Portugal; (ii) co-production for the adaptation and development of new features of a tailored blended care program, and (iii) prototyping, based on quantitative data collected to test usability, quality, understandability and actionability of the resources developed for the program (Figure 1). Utilizing the ADAPT guidance, we used a hybrid approach for intervention development and adaptation [23], with planned adaptations of an evidence-informed intervention to achieve a better fit to the new context and the addition of new components [23,37].

Criteria for mixed-method approaches were followed for this study. The use of the Mixed Methods Appraisal Tool (MMAT) version 2018 provided guidelines for assuring the methodological quality of the study, including specific criteria for a mixed-method design [38].

The involvement of stakeholders was included as an overarching principle central to all stages rather than a discrete stage. Through a person-based approach, an iterative process of data collection was employed to comprehend participants’ views, context, and experiences [39,40]. This insight gathered by involving people with stroke, their families and health professionals from the start and working closely with them was focused on their contribution to a deeper understanding of needs, adaptation and finding new solutions (stage 1). Their involvement was also important during the iterative adaptation and design of new components to inform decisions about content, format, style and delivery (stage 2), which may make a difference in the future engagement and acceptability of the intervention [23,39,41]. The stakeholders’ participation also aimed to gather feedback about intermediate outcomes and to assess usability, quality, understandability and actionability (stage 3).

#### 2.1.1. Stage 1: Evidence Review and Qualitative Study

At the initial stage, we regarded drawing on theory, evidence, and the perspective of the people who will use the intervention as equally important and complementary. A rigorous process of intervention mapping using evidence from existing quantitative primary research, systematic reviews, and meta-analyses was used, taking into account the existence of previous systematic reviews with meta-analyses in the field (e.g., [10,42]). This initial phase of the literature review aimed to identify the most suitable self-management intervention, which was complemented by qualitative research for exploring and understanding the needs, concerns, and motivations of people with stroke and informal caregivers, as well as for gathering the perspective of health professionals about the needs and useful strategies based on their professional experience. Combining both methods, the aim was to provide insight into the potentially effective or cost-effective intervention components from the previous primary and secondary research and provide guidance about the best way to implement it in a particular context [23].

Thus, a literature search was conducted in four databases: Medline, EBSCO, SCOPUS, and ACM Digital by MM and PM. The terms used in the search strategy are described in Supplementary Materials File S1. Afterwards, a multi-perspective qualitative design was used, following a constructivist-interpretivist paradigm and a reflexive thematic analysis approach [43,44,45]. In-depth, individual, and semi-structured interviews were carried out with people with stroke, informal caregivers, and health professionals and were conducted in person or online according to the participant’s preference by MM and DC. The interview guides were pre-tested with three people with stroke, two caregivers and two health professionals, and included initial questions to explore the impact of stroke (for people with stroke and caregivers) and the understanding of self-management (for health professionals), followed by questions to explore perceived needs during home transition, the perception of self-management support provided, and which factors were perceived as facilitators and/or barriers. Then, examples of existing evidence-informed self-management interventions were presented in order to gather participants’ feedback about the relevance of adapting an intervention to promote stroke self-management support in Portugal.

Participants were recruited from three health units, including two hospital stroke units, a primary care center and a community rehabilitation clinic in the district of Setúbal, Portugal. Potential participants were invited in person, by telephone or email, and were purposefully selected considering the study aims by JA, MM and DC. People with stroke were included if they had a confirmed diagnosis of stroke, aged over 18 years, had been discharged from a hospital or inpatient rehabilitation center in the last 2–12 months, had an autonomy level < 4 on the modified Rankin scale at the time of hospital discharge, and were able to understand and commit to the study aims and provide written informed consent. They were excluded if they had a clinical diagnosis of a severe mental or neuropsychiatric disorder that compromised or impaired their ability to participate in the study (e.g., severe depression with psychotic symptoms and/or marked suicidal ideation, decompensated bipolar affective disorder, schizophrenia and other delusional disorders), cognitive impairment (<23 in Mini Mental State Examination), and if they were readmitted to the hospital. Informal caregivers of people diagnosed with stroke were aged over 18 years and identified themselves as the person who assumes most of the care. They were excluded if they had a clinical diagnosis of depression or other neuropsychiatric manifestations that compromised participation in the study and if their relative was readmitted. Health professionals were included if they had a minimum of two years of experience in clinical follow-up or rehabilitation of stroke patients.

#### 2.1.2. Stage 2: Co-Production

In stage two, workshops with an advisory panel composed of members of the research team and key stakeholders, including people with stroke, informal caregivers, academics, and health professionals, were conducted to co-produce the intervention materials and resources. Some participants who were included previously (stage one) were invited to participate, while new members were also invited to join. Findings from stage one and ideas were presented and discussed by all members, feedback on ideas was sought, and refinements were made and presented again until the final content was agreed upon.

Following the person-based approach, the creation of guiding principles, key intervention design objectives and features of the intervention needed was performed by CMP, MM, DC, JMC, and TD and were theoretically underpinned by the social cognitive theory [46,47], which is one of the most widely used models to explain and improve self-management in people with stroke [42]. Moreover, social cognitive theory is frequently used to guide the development of mobile health interventions [28,41].

The development of the digital solution was further guided by an intervention-specific approach, namely the IDEAS framework [41] and was performed by RNM, PM and LPF. Despite numerous frameworks providing guidance on the development of mobile health interventions, most aim to facilitate clinical or patient care rather than modify health behaviors. The IDEAS framework aims to guide the development of digital health interventions to change behavior [41]. The complementary nature of the person-based approach is reinforced within this framework.

#### 2.1.3. Stage 3: Prototyping

In stage three, the draft intervention resources underwent review by potential end users, including people with stroke and health professionals. The testing procedures aimed to assess the usability, quality, understandability and actionability of both a mobile app and a workbook developed. The inclusion and exclusion criteria for both people with stroke and health professionals were similar to those applied in stage one, except for the criteria of time after hospital discharge, which was not considered at this stage. Places and recruitment procedures were also similar to stage one. After presenting a comprehensive study overview in straightforward language to ensure universal participant understanding, informed consent was obtained, and participants completed a detailed sociodemographic questionnaire.

In the subsequent stages of the test protocol of the resource developed for the in-person intervention, participants were introduced to the main content and encouraged to explore freely, providing feedback through the completion of the European Portuguese version of the Patient Education Materials Assessment Tool—for Printable materials (PEMAT-P) form [48,49].

Regarding the test protocols of the developed digital solution, participants were presented with the app functionalities, followed by an interactive phase granting tablet access for specific tasks, designed to assess various user interaction aspects. These tasks included exploring the peers’ stories, Frequently Asked Questions (FAQs), glossary, goal setting, and diary management. Subsequent protocol stages focused on promoting independent exploration, with people with stroke providing feedback through established usability scales, such as the European Portuguese versions of the System Usability Scale (SUS) [50,51] and the User version of the Mobile App Rating Scale (uMARS) [52,53]. Additionally, health professionals contributed to the evaluation process by completing the European Portuguese version of the Patient Education Materials Assessment Tool—for Audiovisual materials (PEMAT-AV) [48,49].

### 2.2. Ethical Considerations

Ethical approval was obtained from the Ethics Commission of the “Centro Hospitalar the Setubal” (Reference n.16/2023F) and the “Unidade Local de Saúde do Litoral Alentejano” (n.018/2023). In accordance with the ethical principles specified by the World Medical Association Declaration of Helsinki, an information document was given to each participant, and details about the purpose, nature and procedures of the study were explained in all project stages. Once all doubts had been answered, participants who agreed to take part in the study gave written consent. The confidentiality and anonymity of the participants were guaranteed using a numerical coding system only known by the research team.

### 2.3. Data Analysis

Qualitative data were transcribed verbatim using Microsoft Office 365—Word and NVivo transcription and analyzed using NVivo 11 (release 1.7.1) software. An inductive thematic analysis was used to identify, explore, and describe patterns of themes through data to gain knowledge from participants’ experiences, thoughts, or behaviors [44,45]. Sociodemographic and clinical data were analyzed using the descriptive statistic measures of mean, standard deviation, median and interquartile range using Microsoft^®^ Excel software for mac, version 16.80.

The reflexive analysis process was guided by a six-phase process for data engagement, coding, and theme development, including data familiarization; systematic data coding; generating initial themes from coded and collected data; developing and reviewing themes; refining, defining, and naming themes and writing the report [44]. Each transcript was coded line by line, firstly from the interview of a person with stroke, with further codes deriving from data sequentially from other people with stroke’s first interviews. These initial steps of coding interviews from the group set “people with stroke” were followed by the development of a coding of themes and sub-themes. Similar steps were conducted within the groups: “caregivers set” and “health professionals set”, providing an initial indication of differences in perspectives. Each interview was coded individually by MM and DC, with frequent debriefing sessions being held with CMP. The subsequent refinement of themes was conducted through discussion and interpretation of the coding three by MM, DC and CMP to increase credibility and ensure rigor and trustworthiness of the analysis process [54].

## 3. Results

### 3.1. Stage 1

The results obtained from the literature search helped the research team to critically reflect on the most suitable self-management intervention. Evidence from previous systematic reviews [10,42,55] and recommendations from clinical guidelines [10,18] about the importance of self-management support based on self-efficacy supported the research team’s option for adapting the Bridges Stroke Self-Management Program (Bridges SSMP). This program is a complex intervention aimed at supporting people with stroke and promoting their ability to self-manage their condition and live more independently [56,57]. Based on self-efficacy and behavior change principles, this intervention was developed and supported by co-production methods, and supports health professionals to integrate self-management into their daily routine of clinical practice. Emphasis is particularly directed toward language employed during interactions with people with stroke, as well as through the effective utilization of self-management tools [58]. Bridges SSMP has been implemented widely across the UK, Ireland, New Zealand, Sweden and Estonia, with good results for implementation, feasibility and acceptability [56,57,59,60,61].

In the qualitative study, a total of 47 participants, including 17 people with stroke, 12 informal caregivers and 18 health professionals, were involved. The majority of people with stroke were male (65%) with an average age of 60.2 ± 12.96 years and time after stroke of 6 ± 3.14 months. Most of the informal caregivers were female (92%), with an average age of 52.58 ± 9.82 years, and reported having no previous experience in care (67%). Health professionals were mostly female (83%), with an average time working with people with stroke of 17.12 ± 8.93 years. Both people with stroke and informal caregivers reported good self-efficacy scores and a medium level of digital and health literacy, as illustrated in Table 1.

#### 3.1.1. Thematic Analysis

**Figure 2:**
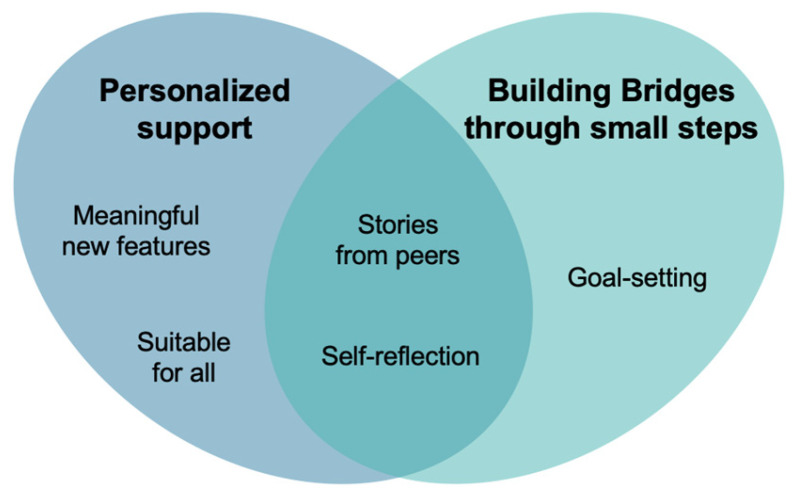
Thematic analysis: themes and sub-themes.

Two major themes were derived from the data analysis: (i) “personalized support” and “building bridges through small steps”, which represented the importance of meaningful information and collaborative strategies to overcome the challenges and difficulties after a stroke. Each theme was connected to sub-themes, as presented in the Venn diagram (Figure 2), and described below in further detail. For each sub-theme, quotes are included with the identification of the participant’s group (PwS—people with stroke; C—caregiver; HP—health professional).

##### Personalized Support

This theme represents the participants’ perspectives about how tailored support may be helpful for both people with stroke and caregivers. The sub-themes: “stories from peers” and “meaningful features” highlight the powerful role of tools, such as stories and individualized data, to empower both people with stroke and caregivers. In common, participants considered that tools should be “suitable for all”, that is, being adaptable to different needs, preferences, and profiles.

- Stories from Peers

The value of contact with stories from people going through similar experiences was perceived as helpful by all participants’ groups. Particularly, people with stroke prioritized stories that had more in common with their own characteristics, experiences, and symptoms. Some participants have reported that having access to stories related to their own experiences could change the way they perceive their ability to respond to emerging needs and challenges. Moreover, some informal caregivers and health professionals emphasized the potential role of the stories in motivating the person with stroke and providing them with useful tools to manage post-discharge experience. They also found it useful to understand the strategies employed by others to overcome challenges after a stroke.

*“Look: “she also felt this like me, or I felt this too. After all, it’s normal”. We feel a little more normal, not so extra-terrestrial. It’s true, that helps.”*

(PwS#05)

*“I spoke to a lot of people who had the same, and I asked them for advice, how they felt afterwards. That helped me a lot”*

(PwS#14)

*“It would be a helpful tool *[referring to the workbook]* both for the person who had the stroke and for the family members who deal more directly with the situation”*

(C#11)

*“I think that having stories from some people who have already gone through the same situation can be a good incentive to help them with their own rehabilitation.”*

(HP#13, Physiotherapist)

The power of stories from others seems to motivate people going through a difficult time. From the participants’ standpoint, stories from peers can serve as a guiding principle for reflecting on the past and consideration of future prospects. However, some participants also emphasized the importance of including stories that may expose the difficulties after stroke, as well as the Portuguese culture and specificities from the Portuguese post-stroke pathways, as reported in the following quotes:

*“People with stroke *[from the workbook]* do not look sick, which is good. However, it may also be important for people to realize that it is common to stay seriously weakened. This may help them to identify themselves with the stories presented”*

(HP#03, Physiotherapist)

*“Other examples, including activities culturally adapted to our Portuguese contexts, such as going to the cafe, going to the supermarket, going for a walk with the dog, will contribute to a better adaptation of the workbook to our culture.”*

(HP#01, Physiotherapist)

- Meaningful New Features

From the perspective of people with stroke, caregivers and health professionals, the potential use of monitoring features focused on health-related data may be useful to support self-management in stroke care. Some participants highlighted the importance of monitoring signs and symptoms (e.g., fatigue, mood levels, blood pressure). Others recognized the relevance of the support for therapeutic management (e.g., therapy sessions, medication). Also, some informal caregivers reported the potential importance of a digital approach in aiding socialization and customizing questions.

*“Older people forget the medication a lot and mix them all the time. I remember that my grandmother used to mix all the medications. Maybe it was quite useful something to manage the medication.”*

(PwS#01)

*“So, if there was an option to do medication management in the app, I think it was important. One more agenda: the user’s agenda for the physiotherapy sessions, to remind exercises to do at home, or a space in the app where the therapist or health professional could put some specific tips.”*

(HP#04, Physiotherapist)

*“For example, it was important to have a page with facts. Or… is not really a chat, but where people could ask questions, and someone could answer. I think that is also important.”*

(C#04)

However, some participants presented a dual perspective on the worth of utilizing a digital tool. Although the option for using a digital tool may be advantageous, it may not fit all. Those who experience greater difficulty and limited digital literacy may prefer having access to the same strategies with a more traditional approach, that is, in-person and paper-based.

*“The disadvantage about the digital solutions, here in Portugal, is that some people do not know how to use technology. These kinds of technologies would be very useful for people, but some don’t know nothing about it. That was visible during the pandemic.”*

(PwS#06)

- Suitable for All

The value of providing strategies depending on the profile, needs, and preferences of the person with stroke and/or family members was highlighted by participants. From their viewpoint, the development of new tools should take into account the age, education level and digital literacy of the potential end users, with different face-to-face or digital strategies to enable access to people with different challenges and circumstances.

*“New technologies are a useful tool, but it really depends on some personal factors. For very old people, who live in rural areas, who do not have access to technology, of course they will not make sense.”*

(HP#14, Social Worker)

*“Would be useful having a blank page *[referring to the workbook]*. Let’s imagine this: there are contacts that may be specific to the region where the patient is hospitalized (…). In addition to national contacts, we could add some local contacts that may be useful.”*

(HP#10, Nurse)

Although participants from across all three groups reported the importance of providing different strategies throughout the recovery process after stroke, the period of planning discharge and transition from hospital to home was perceived as critical. Returning home was characterized as being a period of changes and uncertainties, with participants recognizing difficulties in fulfilling needs and a lack of support from health and social services. Some participants emphasized the importance of providing both tools to support this period, presenting information in a structured manner and making the discharge process more manageable.

*“I think this workbook is very, very, very important for post-discharge. Going home is a dramatic shock.”*

(PwS#05)

*“Really, the only thing I thought it would be important was that when he left [the hospital] he had some support. Because we had still almost two months at home without me having any support.”*

(C#09)

*“It can’t happen a patient leaving the hospital and the family do not knowing how to dress that patient or how to lift the patient, or if the patient doesn’t get up, how to mobilize him.”*

(HP#14, Social Worker)

*“There are several health professionals giving information at the same time, and families can’t understand all… it is the therapist, the social worker, the doctor, the nurse”*

(HP#17, Nurse)

##### Building Bridges through Small Steps

This theme highlights the importance of a collaborative “goal-setting” approach during the recovery process, giving a purpose and motivation to the person with stroke, as well as a better understanding for informal caregivers about their relatives’ evolution. Moreover, “self-reflection” was perceived as an important strategy to enhance self-efficacy through the awareness of accomplishment of small steps and successes.

- Goal Setting

The goal-setting approach was highlighted by participants as a key factor of the proposed tools. On the one hand, health professionals value the possibility of setting goals in collaboration with the person post-stroke, as well as exploring in detail the necessary stages and steps to achieve them. From their viewpoint, the development of a goal-setting feature in the mobile app would also be helpful for both people with stroke and caregivers.

*“The thing I found most interesting in the manual was that part where the person defines goals, I don’t say SMART goals, but almost creating SMART goals for themselves. See goals, let’s define after how long we are waiting for… (…) I think it could be interesting”*

(HP#02, Physiotherapist)

*“I had a patient who had a stroke and came back from vacation showing me a video and saying: “see, I can swim, I can swim in the sea and in the pool.” And he showed me all the videos, so happy. That was our goal since the beginning.”*

(HP#01, Physiotherapist)

On the other hand, setting and building goals in a step-by-step manner was acknowledged as a challenging task by some people with stroke and informal caregivers. Although they recognized its importance and provided examples of doing it intuitively, they suggested that a more structured approach may be beneficial. Some informal caregivers also reported using a step-by-step and personalized strategy to motivate and emphasize the evolution and progression of their relatives.

*“She *[the patient]* was always seeing the glass as half empty, and I sometimes tried to help her see the glass as half full: “You were lying down, you wouldn’t get up, you can already lean against the back of the bed and you can sit up in bed for 5 min without feeling dizzy, without falling backwards”. It was a very progressive and exhaustive process (…). She put a lot of pressure on herself, on the goals she wanted to achieve, and that affected her psychologically”*

(C#05)

*“If people do things just because, they will end demotivated. My mom was always saying: “I can’t, I can’t” *[about communicating through writing]*. I gave her a pen and a paper and I asked her to add carrots to the supermarket list. At first, she couldn’t, but at the second time she was already writing it. I think that the steps to achieve goals will depend on the person, on what is important for her.”*

(C#07)

*“For example, for driving, I set small goals. First, I decided that my goal was to drive to the physiotherapy, which is two minutes distance. I did this for three weeks (…) then, I defined several levels to follow: I increased the distance, going to a commercial surface, then going to Setúbal and I started to broaden my horizons.”*

(PwS#05)

*“It ends up being a motivation to set recovery goals. At this level, perhaps it may be a tool to stop people from giving up, based on the testimonies, some similar and others not so much.”*

(C#06)

- Self-Reflection

This sub-theme represents the perceived role of self-reflection during the collaboration within the triad: person with stroke, caregiver, and health professionals, highlighting its importance for tracking progress over time and gaining confidence to self-manage. From the perspective of health professionals, both tools (workbook and mobile app) may support the person with stroke in tracking their achievements, which can help them reflect on their own progress, avoid frustrations, and promote self-perception, self-efficacy, and motivation. The opportunity to record the goals and achievements was perceived as a valuable resource to promote self-reflection and engage the person with stroke in the recovery process.

*“It gives us a way to encourage the use of strategies for their recovery, to explore reflection or personal reflection.”*

(HP#01, Physiotherapist)

*“Many of them do not have this ability of looking back and reflecting on their recovery. They say: oh, everything is wrong” (…). So, if we go back and look at the records, maybe if I go get to one of these old records and say: -Look here and look now. Can you see the difference? It is palpable.”*

(HP#06, Occupational therapist)

*“I think it may be very interesting and of huge value to invite people to evaluate their own progress. This will help them to set goals and be proactive”*

(C#11)

The main focus of people with stroke was the challenge of reflecting and sharing, which was perceived as possibly difficult to accomplish. Instead of viewing self-reflection as a means to motivate and improve, some participants perceived it as an important way of expressing feelings about the situation they were living in.

*“Thinking about what I’m feeling and what I want to do it is not easy. It is an intimate moment, being at home writing… I am fully aware that I speak openly about what I feel, which helps me. I do not have that fear, but it’s not easy for everyone.”*

(PwS#05)

### 3.2. Stage 2

Considering the findings from stage one, two cycles of four workshops with an advisory panel were held over the course of a six-month period. The advisory panel included four members of the research team and other key stakeholders, including three people with stroke, three informal caregivers, and three health professionals, and aimed to co-produce the intervention materials and resources. The workshops were conducted in the Health School of the Polytechnique Institute of Setúbal, and each was moderated by the coordinator of the project (CMP) and co-moderated by a member of the research team (MM).

The first two workshops aimed to discuss content for the intervention, including the adaptation of the Bridges Stroke Self-Management (SSM) book to the Portuguese context and the development of a mobile app and proposed functionalities informed by findings from the previous stage. The main recommendations from the first two workshops were related to the importance of clarity in the language used in both tools. Contents and specific words were discussed in order to help the text foster self-management principles, inviting the user to perceive both tools as reflective diaries. Moreover, the use of complementary images and videos was also analyzed with the aim of facilitating the understanding of the content. The group suggested using larger and more realistic photos in the layout, as well as brighter colors in the chapters.

The last two workshops were focused on refining ideas and agreements about final contents. Concerning the workbook, decisions were made about the different peer stories (about people with stroke) to be included, and changes were suggested in the chapter: “Questões comuns e dicas” (literally “common questions and tips”). Regarding the mobile app, feedback was given about the number of actions needed to complete tasks and decisions were made about the layouts.

Hence, a final program was co-produced, named ComVida, aiming at empowering people with stroke and their informal caregivers, with the support of health professionals, through an individualized, person-centered self-management approach delivered by trained health professionals. The Bridges self-management training, with accreditation of the Personalised Care Institute, was provided by FJ to 15 Portuguese health professionals, consisting of eight virtual workshops over a six-month period.

**Figure 3:**
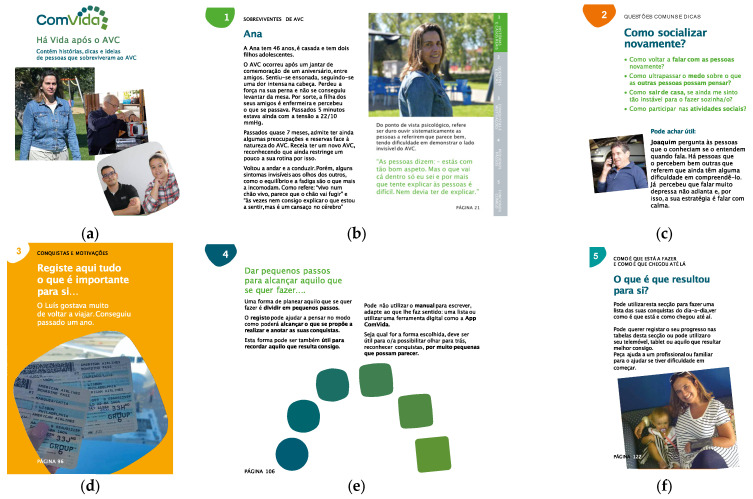
Illustrative examples of the ComVida workbook. (**a**) Cover of the workbook; (**b**) Peers stories: the story of Ana, 46, who suffered a stroke that affected her balance (chapter 1); (**c**) Common questions and tips: “how to socialize again?”, including strategies people with stroke found useful (chapter 2); (**d**) Hopes and ideas, giving examples and space for people to write their hopes for the future (chapter 3); (**e**) Small steps and taking action: set goals (chapter 4); and (**f**) Looking back: with a list of achievements that have worked for Diana (chapter 5).

**Figure 4:**
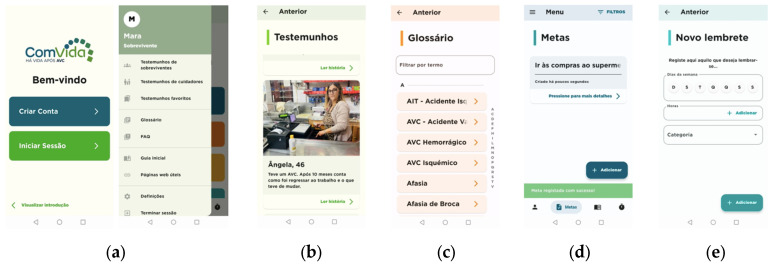
Screenshots of the ComVida app. (**a**) Front page of the app; (**b**) testimonials page; (**c**) glossary page; (**d**) goals page; and (**e**) notifications.

The ComVida program includes a workbook and a mobile app, as illustrated in Figure 3 and Figure 4, respectively. The ComVida workbook aims to support self-management processes through peer support, peer learning, social comparison and modeling, including the experiences and ideas of 15 people with stroke, different ages, stroke severity, symptoms, professional and social reintegration (chapter 1), also showing the recovery strategies they found useful (chapter 2). The workbook aims to support self-reflection also by enabling people to record their own hopes and ideas for the future (chapter 3), plan small steps to take action (chapter 4) and record their meaningful achievements (chapter 5).

The ComVida app was developed using a three-layer architecture to ensure a modular and scalable design, with each layer handling distinct aspects of the system’s functionality. The front end was implemented using Flutter technology [62], which is a framework developed by Google, designed for the creation of mobile applications for both Android and iOS, utilizing Dart as its underlying language. This layer is responsible for implementing the graphical interface of the application and managing interaction logic. The back-end layer encompasses the core logic of the application and all data access services. Developed in Node.JS [63], this component serves as the backbone for the application’s functionality. The implemented database is a non-relational MongoDB [64] database responsible for persisting application data. The data can be broadly categorized into two main groups: (i) Knowledge Base Data that includes narratives, FAQs, glossary, and categories, which can be updated through a web interface; (ii) Data generated by the application and associated with each user, such as user-profiles, reminders, and a diary-entries.

The app included 20 narratives in text, audio or video format, allowing it to be adapted to the needs and profiles of users. It also includes a personal diary, enabling people to (i) record their thoughts, perceived health status and symptoms (e.g., mood, fatigue, physical condition and activity), (ii) add notes, attach images or videos and audio; (iii) add reminders, allowing the person to ensure better medication adherence, manage their medical appointments/treatment sessions, as well as record any other event they want to be reminded of; (iv) understand the meaning of clinical terms in a glossary, including clinical terms frequently used by health professionals (e.g.,: agnosia, aphasia, dyslipidemia, spasticity, among others, in a total of 71 terms); and (i) look at FAQs, such as: “What can I do to reduce the risk of another stroke?”; “I feel tired, is it normal?”; “Can I go back to work?”; “Can I drive again?”; “As a caregiver, how can I prepare home to receive my family member?”, in a total of 15 questions.

### 3.3. Stage 3

A total of 56 participants were included in stage three, including 35 people with stroke (average age: 63.03 ± 10.8 years; average time after stroke: 2.5 ± 5.6 years) and 21 healthcare professionals (average age: 37.8 ± 10.4 years; average professional experience time with people with stroke: 14.5 ± 9.4 years). People with stroke were mostly female (54.3%), married and living with a partner (71.4%), retired (60%) and had less than nine years of schooling (51.4%). Regarding the use of technology, most of them reported using at least a smartphone (74.3%) and using the internet (65.7%), and some mentioned having at least one health app on their smartphone (40%). Most health professionals were female (80.95%) working in hospitals (47.6%). This third stage had the participation of 13 physiotherapists, four speech and language therapists and four occupational therapists.

The results of the usability, quality, understandability and actionality tests are detailed in Table 2.

Regarding the ComVida workbook, both people with stroke and healthcare professionals assessed the understandability, that is, how well the written material was understood, and actionability, that is, how well people can identify what they need to do based on the information provided, with high scores (97% and 100%, respectively), showing a high understandability and actionability of the resource.

**Figure 5:**
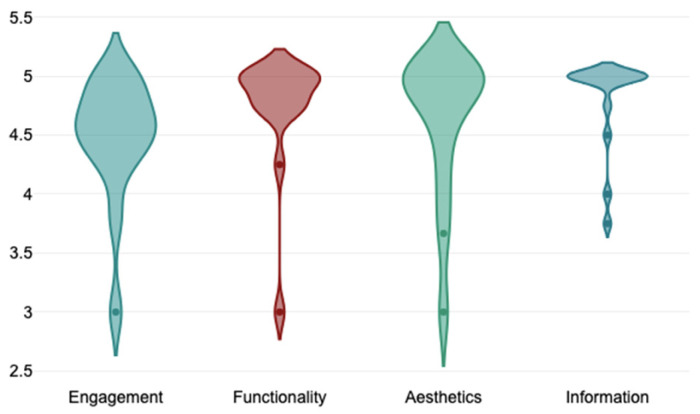
Violin plots of uMARS subdomains of objective quality of the ComVida app.

Concerning the ComVida app, healthcare professionals rated the understandability with similarly high scores (mean 97%). Actionability was rated with slightly lower scores (mean 91%). Moreover, people with stroke rated the usability of the app with an average SUS score of 88.2 points (±14.03), falling within a range of 47.5 to 100. They also rated the quality of the App through the uMARS with a mean total score of 4.61 ± 0.48 on a 5-point Likert scale. In detail, in the assessment of the objective quality of the app, the functionality and information of the domain had the highest ratings (median 4.8, IQR 0.1 for both), followed by aesthetics (median 4.7, IQR 0.1) and engagement (median 4.7, IQR 0.61), as illustrated in Figure 5. The assessment of the subjective quality of the app revealed that most of the participants would recommend this app to people who might benefit from it and rated the overall app with five stars (“one of the best apps I’ve used”) (Table 2).

## 4. Discussion

This study aimed to develop a personalized blended intervention for self-management support after stroke, taking into account the needs of the dyad: people with stroke and informal caregivers. By incorporating the three-stage framework with pragmatic guidance on how to co-produce health intervention [37] and frameworks to develop digital interventions [39,41], a blended program combining in-person and digital solutions was co-produced for the Portuguese context.

Findings from stage one suggest that Portuguese people with stroke may benefit from learning through real-world experiences, strategies recommended by peers, and features addressing specific needs related to record-keeping (including reflections on action and record of small successes of the recovery process), scheduling or medication management. Similar findings were obtained in previous research [59,61] aimed to adapt the Bridges SSMP to other contexts, with participants consistently mentioning how the stories had inspired them or given them ideas on how to approach various situations they encountered during their rehabilitation. Moreover, providing the triad with the opportunity to implement a collaborative goal-setting approach and set meaningful goals was pointed out by participants as highly useful in supporting self-management during the rehabilitation process. Previous findings also highlight the importance of focusing on meaningful goals and working in partnership with trusting, supportive relationships to contribute to building self-confidence and a sense of self-worth [65,66,67]. However, other Portuguese studies revealed difficulties in the involvement of both people with stroke and informal caregivers during the decision-making throughout rehabilitation [12], which emphasizes the importance of the development of the ComVida intervention.

Regarding the format of solutions, participants highlighted the importance of designing resources tailored to different profiles of end users and giving the option to use an in-person, a digital or both approaches combined when needed or preferred by both people with stroke and caregivers. Combining personalized in-person interventions with the use of digital approaches has been shown to be of preference by people with stroke in previous research [29]. Both may have the potential to create bridges between people with stroke, caregivers, and health professionals.

By using a co-production approach and an iterative process, the development of the ComVida resources during the workshops resulted in a wealth of insights and enriched tools, which are expected to better respond to the needs of potential end users and ensure greater acceptability of the tools in the future [58,68]. The advisory panel was participatory and collaborative, and all members were provided with opportunities to input and share their ideas. The goal was to merge the participants’ perspectives about the resources to be used by people with stroke, informal caregivers, and health professionals. Toward that end, a comfortable environment was established where representative end users were heard, and opinions were taken into consideration [69]. All participants demonstrated a high level of interest, recognizing the added value of the topic and resources for the development of a blended self-management intervention and provided recommendations for improvement. For the research team, the co-production process also represented a learning process, confronting scientifically grounded assumptions about self-management after a stroke. Being open to this learning process was indispensable and allowed collaboration with the study participants on eye level during the sequential stages.

The assessment of the ComVida tools showed that both are well-accepted among people with stroke and health professionals. The SUS mean score falls within the 85th to 89th percentile, corresponding to an A-grade, which is indicative of a “Good” usability score [51]. This result was supported also by the scores obtained in the uMARS, with a five stars global rating score. These results were superior to scores obtained from another Portuguese health app, whose ratings were inferior to two stars [70] and other mobile apps developed for different contexts (e.g., [71,72]). Although the functionality, information content, aesthetics, and engagement of the app were well appreciated (all with uMARS scores above 4.5), further improvements are required. The inclusion of other topics of interest in FAQs from the perspective of end users and the improvement of navigation (actions needed) through the goal-setting process are examples of future improvements to consider by the research team.

As practical implications, these findings indicate the potential for successful implementation of a person-centered and tailored blended approach to support post-stroke self-management. The relevance of this approach to patient empowerment, motivation, and development of self-management skills that enable an active engagement in rehabilitation was outlined both by participants from the study and previous research (e.g., [10]). This is expected to foster a better anchoring of patients’ and families’ needs in the rehabilitation process, reducing the risk that the health professional is the one who defines the rehabilitation goals [12,67].

As limitations of the study, we acknowledge the potential for bias in results due to the use of purposive samples, where participants may have had a greater predisposition toward the use of mobile health solutions. Nevertheless, the findings highlight the considerable potential of the ComVida solution. It demonstrates its capacity to foster behavior change and empower Portuguese people with stroke during the recovery process. Moreover, there are limitations with the generalizability of the study findings to a broader context. The study was undertaken in the context of transition to home and adjustment following a stroke in a Portuguese context. Attempts were made to give the reader the possibility of considering its transferability to other Portuguese and international contexts. However, regional and cultural differences should be taken into consideration.

Implications for further research can comprise specific tools for informal caregivers to better suit their needs and preferences. Then, a small-scale evaluation could aim to evaluate a signal of the efficacy of ComVida on self-efficacy and behavior change of both people with stroke and informal caregivers/families. These results would make ground for evaluating the effectiveness of the ComVida solution on self-efficacy, quality of life, mood, impact of stroke and functional capability, as well as on community reintegration.

## Figures and Tables

**Table 1:** Participant sociodemographic and clinical/professional characteristics.

Population	Outcome	Results
People with Stroke (*n* = 17)	Gender (male)	11
	Age (years: Mean ± SD; Min–Max)	60.2 ± 2; 34–80
	Time after stroke—months (Mean ± SD; Min–Max)	6 ± 3.1; 2–11
	Regular use of technology (yes)	7
	GSE scale (Mean ± SD; Min–Max)	29.2 ± 4.6; 20–38
	SIS 3.0 (Mean ± SD)	52.1 ± 15.6; 25.2–74.2
	e-Heals (Mean ± SD; Min–Max)	25.8 ± 9.5; 5–37
	HLS-EU-PT (Mean ± SD; Min–Max)	34 ± 11.9; 4.8–49.5
Informal caregivers (*n* = 12)	Gender (female)	11
	Age (years Mean ± SD; Min–Max)	52.6 ± 9.8; 36–69
	Laboral Situation (Employee)	10
	Regular use of technology (yes)	10
	Previous experience of caring (no)	8
	QASCI-vr (Mean ± SD; Min–Max)	2.9 ± 0.4; 2.4–3.5
	GSE scale (Mean ± SD; Min–Max)	33.0 ± 3.6; 27–40
	e-Heals (Mean ± SD; Min–Max)	27.4 ± 7.1; 12–37
	HLS-EU-PT (Mean ± SD; Min–Max)	36.9 ± 7; 26.1–47.9
Health professionals (*n* = 18)	Gender (female)	15
	Working experience with PwS-years (Mean ± SD; Min–Max)	17.2 ± 8.9; 5–30
	Professional setting (*n*)	Hospital (inpatient stroke unit and outpatient rehabilitation): 15
		Primary health care: 1
		Community rehabilitation: 2
	Profession (*n*)	Physiotherapist: 7
		Speech and language therapist: 2
		Occupational therapist: 2
		Medical doctor: 1
		Psychologist: 1
		Social worker: 1
		Nurse: 4

SD-standard deviation; Min-minimum; Max-maximum; GSE—General Self Efficacy Scale; SIS—Stroke Impact Scale; e-Heals—eHealth Literacy Scale; HLS-EU-PT—European Health Literacy Survey—Portuguese version; QASCI-vr—Informal Caregiver Burden Assessment Questionnaire—short version; PwS—People with Stroke.

**Table 2:** Assessment of the ComVida tools.

Tool	Outcome	Results		
		Total Score	Score PwS	Score HP
Workbook ComVida	PEMAT-P			
	Understandability (%)	97.4%	95.8%	98.9%
	Actionality (%)	100%	100%	100%
	PEMAT-AV			
	Understandability (%)	-	-	96.8%
	Actionality (%)	-	-	90.8%
	SUS Average (Mean ± SD)	-	88.2 ± 14.03	-
Mobile App ComVida	uMARS total score (Mean ± SD)	-	4.61 ± 0.48	-
	Objective quality of the App (Mean ± SD)	-	4.66 ± 0.49	-
	Subjective quality of the App (Mean ± SD)	-	4.57 ± 0.53	-
	Subjective quality of the App	-	5 stars (1–5)	-

PEMAT-P = Patient Education Materials Assessment Tool—for Printable materials; PEMAT-AV = Patient Education Materials Assessment Tool—for Audiovisual materials; uMARS = Mobile App Rating Scale-user version; SUS = System Usability Scale; PwS = People with stroke; HP = Health professionals.

## Data Availability

Data are contained within the article.

## Supplementary Materials

The following supporting information can be downloaded at https://www.mdpi.com/article/10.3390/jcm13010300/s1, File S1: Search strategies.
