# Supplementary material for: Building Bridges between People with Stroke, Families, and Health Professionals: Development of a Blended Care Program for Self-Management
Source: J Clin Med. 2024 Jan 4;13(1):300. doi: 10.3390/jcm13010300 (PMC10779663; doi:10.3390/jcm13010300)
Supplement: Supplementary file 1 [file jcm-13-00300-s001.zip › jcm-2806175-supplementary.pdf]

Supplementary material File S1

Database: MEDLINE

Search strategy:

1. (((("cerebrovascular disorders"[All Fields]) OR ("basal ganglia cerebrovascular disease"[All Fields])) OR ("brain ischemia"[All Fields])) OR ("stroke"[All Fields])) OR (brain infarction)
2. (((((stroke[Text Word]) OR (poststroke[Text Word])) OR (post-stroke[Text Word])) OR (cerebrovasc*[Text Word])) OR (brain vasc*[Text Word])) OR (cva[Text Word])
3. ((((((((brain*[Text Word]) OR (cerebell*[Text Word])) OR (intracran*[Text Word])) OR (intracerebral[Text Word])) OR (ischemi*[Text Word])) OR (emboli*[Text Word])) OR (thrombo*[Text Word])) OR (embolis*[Text Word])) OR (occlus*[Text Word])
4. (hemiplegia) OR (paresis)
5. (((hemipleg*[Text Word]) OR (hemipar[Text Word])) OR (paresis[Text Word])) OR (paretic[Text Word])
6. ((brain injuries) OR ("brain injury, chronic"[MeSH Terms]))
7. gait disorder, neurologic[MeSH Terms]
8. ((((((#1) OR (#2)) OR (#3)) OR (#4)) OR (#5)) OR (#6)) OR (#7)
9. (self efficacy[MeSH Terms]) OR (self care[MeSH Terms])
10. ((self administration[MeSH Terms]) OR (self assessment[MeSH Terms])) OR (self concept[MeSH Terms])
11. ((((patient compliance[MeSH Terms])) OR (patient education as topic[MeSH Terms])) OR (patient participation[MeSH Terms])) OR (patient satisfaction[MeSH Terms])
12. (consumer health information[MeSH Terms]) OR (consumer information[MeSH Terms])
13. ((((attitude to health[MeSH Terms]) OR (health behavior[MeSH Terms])) OR (health education[MeSH Terms])) OR (health knowledge, attitudes, practice[MeSH Terms])) OR (health promotion[MeSH Terms])
14. ((life style[MeSH Terms]) OR (disease management[MeSH Terms])) OR (risk reduction behavior[MeSH Terms])
15. ((((adaptation, psychological[MeSH Terms]) OR (motivation[MeSH Terms])) OR (goals[MeSH Terms])) OR (problem solving[MeSH Terms])) OR (decision making[MeSH Terms])
16. (((((((self care[Text Word]) OR (self-care[Text Word])) OR (self management[Text Word])) OR (self-management[Text Word])) OR (self efficacy[Text Word])) OR (self-efficacy[Text Word])) OR (self monitor*[Text Word])) OR (self-monitor*[Text Word])
17. ((((((((((self[Text Word]) OR (oneself[Text Word])) OR (care[Text Word])) OR (educat*[Text Word])) OR (participat*[Text Word])) OR (behaviour[Text Word])) OR (behavior[Text Word])) OR (compliance[Text Word])) OR (centered[Text Word])) OR (goal settings[Text Word])) OR (copping[Text Word])
18. ((((((((#9) OR (#10)) OR (#11)) OR (#12)) OR (#13)) OR (#14)) OR (#15)) OR (#16)) OR (#17)
19. Randomized Controlled Trial
20. controlled Clinical Trials
21. control groups

22. (clinical trials) OR (research design)
23. ((random*[Text Word]) OR (RCT[Text Word])) OR (RCTs[Text Word])
24. (controlled trial*[Text Word]) OR (controlled stud*[Text Word])
25. (((((#19) OR (#20)) OR (#21)) OR (#22)) OR (#23)) OR (#24)
26. ((#8) AND (#18)) AND (#24)
27. ((#8) AND (#18)) AND (#24)
28. ((#8) AND (#18)) AND (#24)
29. ((#8) AND (#18)) AND (#24)

Database: EBSCO

Search strategy:

1. "Cerebrovascular disorders"OR "basal ganglia cerebrovascular disease" OR "brain ischemia" OR "stroke" OR " brain infarction"
2. Stroke OR poststroke OR cerebrovasc* OR brain Vasc* OR cva
3. Hemipleg* OR hemipar* OR paresis OR paretic
4. "brain injuries" OR "brain damage, chronic"
5. "gait disorders, neurologic"
6. "gait disorders, neurologic+"
7. S1 OR S2 OR S3 OR S4 OR S5 OR S6
8. 7 AND Limiters – Date published: 20160101-20221231
9. "self-efficacy" OR "self care"
10. "self administration" OR "self assessment" OR "self concept"
11. "patient satisfaction" OR " patient education" OR "patient compliance"
12. "consumer health information"
13. "attitude to health" OR "Health behavior" OR "health education" OR "health knowledge" OR "health promotion"
14. "life style" OR "disease management"
15. Adaptation, psychological OR motivation OR (goals and objectives) OR problem solving OR decision making
16. Health plan implementation
17. (self-care OR self-care OR self management OR self-management) Or self efficacy OR self-efficacy
18. S9 OR S10 OR S11 OR S12 OR S13 OR S14 OR S15 OR S16 OR S17
19. Randomized controlled trials
20. Systematic review
21. Meta-analysis
22. Study design

23. Program evaluation
24. S19 OR S20 OR S21 OR S22 OR S23
25. S24 AND S18 AND S8

Database: SCOPUS

Search strategy:

(TITLE-ABS-KEY (stroke OR poststroke OR post-stroke OR cerebrovasc* OR brain vasc* OR cerebral vasc* OR cva* OR apoplex* OR sah OR brain* OR cerebr* OR cerebell* OR intracran* OR intracerebral OR isch?emi* OR infarct* OR thrombo* OR emboli* OR occlus* OR haemorrhage* OR hemorrhage* OR haematoma* OR hematoma* OR bleed* OR hemipleg* OR hemipar* OR paresis OR paretic) AND TITLE-ABS-KEY (self AND care OR self-care OR self AND managementor AND self-management OR self AND efficacy OR self-efficacy OR self AND monitor* OR selfmonitor* OR educat* OR participat* OR behaviour* OR behavior* OR compliance OR centered OR goal* OR decision* OR coping) AND TITLE-ABS-KEY (control OR treatment OR experiment* OR intervention AND quasi-random* OR quasirandom* OR pseudo-random* OR pseudo AND random* OR treatment OR therapy OR procedure OR manage*))

Database: ACM Digital

Search strategy:

[[[All: stroke] OR [All: "cerebrovascular disorders"] OR [All: poststroke]] AND [[All: self-management] OR [All: "patient education"] OR [All: "behavio?r change"] OR [All: "disease management"]] AND [[All: "randomized controlled trials"] OR [All: "effect random* "] OR [All: controlled trial*" "controlled stud*] OR [All: " rct effectiveness effectivity) AND [[All: digital] OR [All: ehealth] OR [All: mhealth] OR [All: virtual] OR [All: telehealth]]] OR [All:))]
